# Supplementary material for: Learning curve and surgical outcome of robotic assisted colorectal surgery with ERAS program
Source: Sci Rep. 2022 Nov 29;12:20566. doi: 10.1038/s41598-022-24665-w (PMC9709162; doi:10.1038/s41598-022-24665-w)
Supplement: Supplementary file 2 — Supplementary Information 2. [file 41598_2022_24665_MOESM2_ESM.zip › RAL Raw data-Ñ[▒K/095/OP N_ñw╝╨░O▒Kñσ.pdf]

W52 / W52

臺中榮民總醫院
手術室護理記錄

[redacted]

基本資料

- 室別： 開刀房-17室，手術科別： CRS，手術類別： 常規，日期： 2021/07/01
- 進入時間： 1505 ； 麻醉開始時間： 1520 ； Time Out時間： 1610 ； 劃刀時間： 1611 ； 手術結束時間： 2040 送出時間： 2100 ；
- 麻醉方式： GE
  診斷：rectum ca 4 cm 6" s/p SCRT
  手術名稱：1.Robot Assisted Low Anterior Resection2.Ileostomy
  手術局部用藥：NIL
  術前預防性抗生素：
  - 抗生素名稱一： Veterin 1GM IVD/由麻醉護理人員給予
  - 時間： 1555
  - 上次簽章: 林家羽

  ，追加預防性抗生素：
- 手術醫師： 林俊余，林陳彥，陳映菁，郭達雄
- 麻醉醫師： VS 齊光彪
-
  - 刷手護士A ： 王明盈，時間： 1505，接替刷手一： 邱儷嫖，時間： 1800
  - 流動護士A ： 林家羽，時間： 1505，流動護士B ： 歐真勤，時間： 1505，接替流動一： 許臺真/林家羽，時間： 1730/1800，接替流動二： 陳家瑋/林家羽，時間： 1900
- 更多接替及流動護士： 接替流動三： 陳家瑋/彭芳渝，時間： 2000

皮膚狀況

A-V Shunt： 無
術前： 完整
記錄者： 上次簽章: 林家羽
術後： 完整
記錄者： 彭芳渝

手術準備

手術臥式 單一手術臥式

-
  - 手術臥式： 仰臥
  - 使用附件： 溫毯 0 ℃，頭圈，墊肩，其它： 腳架ｘ２、腳踝墊X2、Silicone padx1
  - 皮膚準備： 10%Aq Betadine，Hibiscrub，2% Chlorhexidine
  - 電燒： 使用電燒: 電極片位置： 其它： 電燒墊，記錄者： 上次簽章: 林家羽

止血帶T： 無

植入物

- 植入物名稱/號碼/數量/部位：stapler 45green *2/rectum

，記錄者： 上次簽章: 林家羽

標本送檢

- 細菌培養 無
- 細胞學 無
- 冰凍切片 無
- 病理標本
  組織名稱一：Distal cut end
  ,
  組織名稱二：IMA root
  ,
  組織名稱三：rectum
- 截肢 無

標本交付 標本： 1 袋 ， 標本： 0 盒 ， 標本： 2 瓶 ， 細胞學： 0 管 ， Culture Tube ： 0 管 ， 被交付醫師： R 郭連雄 ，

- 記錄者： 上次簽章: 林家羽
- 時間： 1950

傷口術後敷料

傷口敷料：

- 請於圖上標示部位：

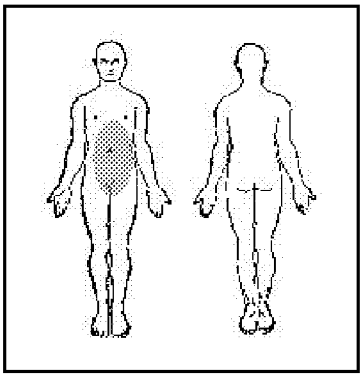

- 傷口敷料： 軟性敷料

導管

導管	天數	導管資訊	插入日期	插入時間	更換日期	拔除日期	拔除時間	拔除原因	預計拔除/更換	預計拔除/更換日期
Foley(1)	1	號碼:16 Fr.; 部位:膀胱; 註記:10cc;	2021/07/01	1540						
Penrose Drain(1)	1	號碼:12 mm. mm.; 部位:骨盆腔;	2021/07/01	1930						
Double Lumen CVC(1)	1	號碼:8Fr ; 部位:; 象限:LU; 註記:15cm;	2021/07/01	1530						
A-line (1)	1	號碼:20G ; 部位:左手;	2021/07/01	1515						

用物計數

- 手術中出血量： minimal c.c.
- 手術中尿量： 250 c.c.
-
    - 其它： 無

用物計數： 多切口同時縫合

- 1.器械： 正確 ，2.縫針： 正確 ，3.刀片： 正確 ，4.紗布： 正確 ，5.腹部墊： 未使用 ，6.紗球： 未使用 ，7.PEANUTS ： 未使用 ，8.紗條： 未使用 ，9.絲帶： 未使用 ，10.針頭： 未使用 ，11.Vessel loop： 未使用 ，12.Boots： 未使用 ，13.Jelco： 未使用 ，14.棉條： 未使用 ，
    - 第一次：
        - 刷手護士 ： 邱欐媃
        - 流動護士： 上次簽章: 林家羽
        - 時間： 1950

,

    - 第二次：
        - 刷手護士 ： 邱欐媃
        - 流動護士： 彭芳渝
        - 時間： 2010

,

    - 第三次：
        - 刷手護士 ： 邱欐媃
        - 流動護士： 彭芳渝
        - 時間： 2015

備註

備註： R17-1 davinic 10mm0度

- 記錄者： 上次簽章: 林家羽
- 時間： 1505

填寫護理人員 彭芳渝

正式摘要

W52 / W52

doctorRank:B
